# Targeting Hippo-YAP/TAZ signaling pathway: an updated review demonstrating the therapeutic potential of key plant derived anticancer compounds

**DOI:** 10.3389/fphar.2025.1743102

**Published:** 2026-01-21

**Authors:** Deena Elsori, Pratibha Pandey, Kholoud Alshaikh, Ali G. Alkhathami, Mohd Saeed, Ajay Singh, Fahad Khan

**Affiliations:** 1 Faculty of Resilience, Rabdan Academy, Abu Dhabi, United Arab Emirates; 2 Centre for Research Impact and Outcome, Chitkara University, Rajpura, Punjab, India; 3 Department of Biological Sciences, Faculty of Science, King Abdulaziz University, Jeddah, Saudi Arabia; 4 Department of Clinical Laboratory Sciences, College of Applied Medical Sciences, King Khalid University, Abha, Saudi Arabia; 5 Department of Biology, College of Science, University of Hail, Hail, Saudi Arabia; 6 School of Applied and Life Sciences, Uttaranchal University, Dehradun, India; 7 Department of Community Medicine, Saveetha Medical College and Hospital, Saveetha Institute of Medical and Technical Sciences, Chennai, Tamil Nadu, India

**Keywords:** anticancer, cancer therapy, hippo signaling pathway, natural compounds, YAP/TAZ

## Abstract

A signaling mechanism that has persisted through evolution, the Hippo pathway is involved in the development and progression of many different types of cancer. Specifically, the complex comprising YAP, TAZ and TEAD is a crucial component of the Hippo signaling, which governs cell growth and stem cell activity. The upregulation of YAP/TAZ/TEAD complex has been demonstrated to result in cellular proliferation, transformation, and ultimately, carcinogenesis. Consequently, it has been shown that Hippo signaling is a prospective target for cancer treatment and prevention. Numerous natural compounds have been identified as inhibitors of the Hippo signaling pathway that downregulate YAP and TAZ in various ways. In several cancer models, plant-derived natural compound inhibitors have been shown to function as both preventative and therapeutic agents. This study examined the modulatory effects of extensively investigated antitumor natural products on the Hippo signaling system and highlights new advancements in Hippo signaling inhibitors that enhance the efficacy of standard cancer therapies. This article offers extensive insight into plant derived anticancer compounds mainly apigenin, curcumin, EGCG, resveratrol, homoharringtonine, and ursolic acid of the Hippo pathway, specifically YAP/TAZ, in several cancer therapies. This will enhance the discovery of novel Hippo inhibitors and the optimal therapeutic application of Hippo signaling-related pharmaceuticals in synergistic cancer therapies.

## 1 Introduction

The hallmark of cancer is the existence of cells that are rapidly and continuously dividing. In the past, conventional therapies such as surgery and chemotherapy were the only methods to treat cancer. However, the identification of tumor suppressor genes and oncogenes has led to the idea that drugs or pharmaceuticals can be used to target individual proteins for cancer therapy (Senga and Grose, 2021). Recent progress in emerging technologies such as next-generation sequencing and multi omics analysis has elucidated the intricate relationship of signaling pathways that create complex networks within cells that, when comprehensively mapped, can be used for enhanced targeted therapeutics (Yip and Papa, 2021). Over the past 20 years, many researchers have come to understand the importance of Hippo signaling. This attempt included searching for the mammalian equivalents of this pathway and how they interact with other important signaling cascades that play a crucial role in cell proliferation and survival. Furthermore, the association of Hippo signaling with the regulation of intracellular and extracellular signals was explored, where potential therapeutic interventions could be targeted (Fu et al., 2022).

The Hippo signaling is a very well-preserved pathway that helps cells grow, die, and change into other types of cells. It gets its name from the protein kinase Hippo (Hpo), which is its key part. It signifies one of the latest identified routes that restrict organ size (Harvey et al., 2013; Guo et al., 2025). One of the more well-known pathways is the Salvador-Warts-Hippo signaling cascade, which contain Yes-associated protein (YAP) and transcriptional co-activator with PDZ-binding motif (TAZ) (Harvey and Tapon, 2007). A multitude of cellular functions, tissue homeostasis, and the growth of organs in the balanced condition are all dependent on the YAP/TAZ complex (Mokhtari et al., 2023; Hillen et al., 2024). Nevertheless, it has been demonstrated that these transcriptional coactivators serve as important tumorigenic drivers that facilitate migration, boost cellular proliferation, inhibit apoptosis, and connect extracellular signals to gene expression programs. Consequently, YAP and TAZ offer a significant opportunity for the development of treatments aimed at this pivotal pathway (Ghaboura, 2025; Talukdar et al., 2025).

Attempts have been made to develop novel agents from plants to prevent cancer metastasis and invasion (Zhang et al., 2024; Wang et al., 2023). Nevertheless, due to concomitant toxicity, adverse side effects, and diminished selectivity and specificity, few have received approval. Consequently, there is an increasing need to investigate innovative anticancer agents to combat cancer (Abdellatef et al., 2022). The recent identification of naturally occurring phytochemicals with significant anticancer properties found in food-based diets has led to the development of chemotherapy regimens that use these compounds alongside conventional chemotherapy drugs. It has been suggested that approximately one-third of malignancies may be averted by dietary regulations and consistent physical exercise (Farahmand et al., 2017).

Moreover, numerous obstacles related to the efficient and secure application of these natural phytochemicals have been surmounted by innovative methodologies employed in the pharmaceutical sector (Atanasov et al., 2021). Natural substances that have been shown to fight cancer in the past are now great candidates for anticancer medications since they are less toxic and work well against many types of cancer (Dehelean et al., 2021; Hołota and Posmyk, 2025).

Among these widely recognized phytochemicals are apigenin, curcumin, resveratrol, green tea polyphenols, soy isoflavones, and artemisinin; they are all members of the class of bioactive substances known as “phytochemicals” (Rai et al., 2025). Thus, diets containing various phytochemicals have been suggested to yield more pronounced effects, and may serve as a primary defense against cancer. Conversely, certain phytochemicals, such as curcumin, can confer DNA protection against damage during radiotherapy and offer a prolonged protective effect. Studies have indicated that phytochemicals, when used independently and not in conjunction with chemotherapies, can exhibit anticancer properties (Sohel et al., 2023). These distinctive effects are partially attributed to their inhibitory impact on the Hippo signaling, which regulates epithelial-mesenchymal transition (EMT), the proliferation of cancer stem cells (CSCs) and metastasis (Huang et al., 2020; Ordaz-Ramos et al., 2023).

Moreover, a growing body of research has examined the anticancer activity of many natural plant-derived substances in relation to their potential functional interactions with Hippo components (You et al., 2022; Bala et al., 2025). A significant number of these studies have indicated that phytochemicals influence the activities of Hippo components, including YAP and TAZ, and their associated anticancer effects, suggesting substantial promise for these drugs in future targeted cancer therapies. Given the scarcity of thorough reviews on this subject, the current study aimed to elucidate the Hippo-related effects of anticancer plant-derived molecules and their prospective applications in cancer therapy (Moloudizargari et al., 2022). The objective of this review is to elucidate the Hippo pathways implicated in cancer development, followed by an examination of the diverse effects of phytochemicals with significant modulatory properties on this particular pathway associated with cancer development, stemness, and metastasis.

## 2 An overview of hippo signaling pathway

Similar to other intercellular signaling pathways, the majority of the components and fundamental signal transduction mechanisms linked to Hippo signaling were initially found in fruit fly, or *Drosophila*, and subsequently demonstrated to be conserved in other animal species. This pathway has been the subject of increasing research over the last 15 years because of its important regulatory effects on growth, regeneration, and cancer-promoting functions (Misra and Irvine, 2018).

The Hippo pathway in mammals include YAP, mammalian Ste20-like kinase 1/2 (MST1/2), sav family WW-domain containing protein 1 (SAV1), large tumor suppressor 1/2 (LATS1/2), and/or its counterpart TAZ, which is encoded by the WW domain-containing transcription regulator 1 (WWTR1) gene. The striatin (STRN)-interacting phosphatase and kinase (STRIPAK) complex typically functions upstream of kinase kinase kinases (MAP4Ks) and MST1/2, thereby inhibiting the Hippo signaling pathway (Bae et al., 2017; Chen et al., 2019). In contrast, activation of the Hippo signaling involves MST1/2, MAP4Ks, and its scaffold protein SAV1, which phosphorylate LATS1/2 and its scaffold MOB1A/B (Zheng et al., 2015). Following activation, LATS1/2 phosphorylates and inhibits YAP and TAZ, thereby obstructing their movement into the nucleus for interaction with TEAD 1–4 (Badouel and McNeill, 2011). MST1 and MST2 are serine/threonine kinase whose activity is augmented when they bind to the scaffold protein SAV1 *via* their C-terminal domains (Sav/Rassf/Hpo) (Yu and Guan, 2013). MST1/2 also promotes the interaction between MOB1A/B and LATS1/2. Recent findings indicate that WWC proteins facilitates this process by acting as scaffold proteins (Qi et al., 2022). Moreover, apart from MST1/2, several proteins in the MAP4K family have been implicated in the stimulation of LATS1/2 (Meng et al., 2015; Yang et al., 2024). Thus, MST1/2 or MAP4K proteins stimulation initiates the Hippo signaling. The simultaneous depletion of these protein significantly obstructs downstream signaling (Plouffe et al., 2016). MOB1A/B performed two distinct roles in activation of Hippo signaling. The first role is to facilitate the interaction of MST1/2 and LTS1/2 by serving as a scaffold. Furthermore, modified MOB1A/B promotes the stimulation of LATS1/2 by causing a conformational alteration in LATS (Ando et al., 2021; Praskova et al., 2008). LATS1/2 are serine/threonine kinases belonging to the AGC kinase family. They engage in direct interaction with YAP/TAZ after stimulation. WW domains on LATS1/2 have been proposed as potential mediators of this interaction (Furth and Aylon, 2017; Moroishi et al., 2016). Strong evidence indicated that activated LATS1/2 phosphorylate and deactivate YAP and TAZ, the primary downstream regulators of the Hippo signaling (Basak and Ain, 2022).

**FIGURE 1:**
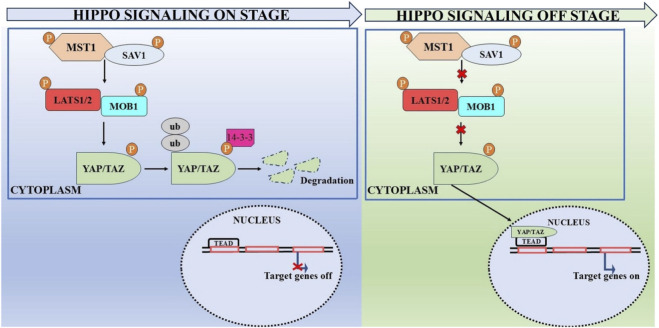
An overview of the Hippo signaling pathway in activated and non-activated state.

In response to stimulation of the Hippo pathway, YAP/TAZ activity is downregulated which further resulted in YAP/TAZ degradation in the cytoplasm. In contrast, when Hippo signaling is inhibited, unphosphorylated YAP/TAZ migrates to and aggregates in the nucleus, where they engage with TEA domain (TEAD) transcription factors to modulate target gene expression and promote cell proliferation and survival (Kim et al., 2023; van Soldt and Cardoso, 2020) (Figure 1).

Numerous context-dependent functions of YAP have been documented and examined (Cunningham and Hansen, 2022). It has been demonstrated that YAP/TAZ interacts with several transcription factors, including p73, RUNT-related transcription factors (RUNX1 and RUNX2), SMAD1, SMAD2/3, SMAD7, and T-box transcription factor 5 (TBX5) (Boopathy and Hong, 2019). Additional studies of Hippo and TGF-β interactions suggest that TAZ/YAP can influence TGF-β receptor activation and enhance nuclear retentivity (Labibi et al., 2020). In response to stress, the critical energy indicator component AMP-activated protein kinase (AMPK) promptly phosphorylates and suppresses YAP and TAZ by inhibiting their association with TEAD (Koo and Guan, 2018). Notch signaling pathway is also regulated by YAP/TAZ mediated upregulation expression of Jagged1 (Kim et al., 2017). YAP/TAZ also identified as a constituent of a deleterious complex within the Wnt/β-catenin pathway. The nuclear translocation of YAP/TAZ resulted in β-catenin mediated upregulation of various target genes, which collaboratively promotes cell growth and tumor progression (Yang D. et al., 2021; Rosenbluh et al., 2012).

It is widely recognized that TEADs are important element of YAP/TAZ action. TEAD possesses a “TEA” DNA binding site that interacts with the target genes stimulators, as well as a YAP/TAZ binding site that connects with transcription associated cofactors. The phosphorylation at cysteine residues of N- and C- terminal site has been demonstrated to impact the stability of TEADs and expression of target genes (Noland et al., 2016; Chan et al., 2016). Stress induced p38/mitogen activated protein kinase (MAPK) has been shown to have significant impact on the nucleocytoplasmic shuttling of TEADs (Lin et al., 2017). Vestigial-like family member 4 (VGLL4) inhibits YAP activity by obstructing its TEAD binding site. T-cell lymphoma invasion and metastasis 1 (TIAM1) interrupts the YAP/TAZ-TEAD interaction by interacting with YAP/TAZ (Yamaguchi and Taouk, 2020). In response to Hippo pathway downregulation, YAP/TAZ migrates to nucleus and binds to TEADs, activating target genes such as cysteine rich protein 61 (CYR61), AXL, and CTGF. Activation of these gene resulted in augmented cell proliferation and development (Allegra et al., 2021; Choi and Kim, 2024).

## 3 Hippo signaling pathway and tumorigenesis: from the lens of YAP/TAZ and TEAD

The Hippo signaling pathway is frequently dysregulated in various cancer types; however, mutations in this pathway are comparatively uncommon. Inactivation of components of the core kinase module occurs through mutations in prevalent cancer types, but these events are rare and typically found in less than 10% of cancer cases (Wang et al., 2018; Li and Guan, 2022). The direct role of the pathway in initiating and advancing cancer is evident in animal models, where YAP/TAZ hyperactivity induces various cancer types and metastasis, as well as in certain rare human cancers characterized by frequent loss-of-function mutations and deletions of kinase module genes (Table 1).

**TABLE 1:** Association of various targets of Hippo signaling pathway including YAP, TAZ, MST and LATS in multiple carcinomas.

Cancer	Cancer model	Key component (s)	Modulatory effect	References
Breast cancer	*In vitro*, *in vivo*	TAZ, MST and LATS	Stimulated self-renewal and tumor-initiation capacities	Cordenonsi et al. (2011)
	*In vitro*, clinical samples	TAZ	Association with EMT and metastasis	Díaz-Martín et al. (2015)
	*In vitro*, *in vivo*	YAP/TAZ	Stimulated self-renewal and tumor-initiation capacities	Kim T. et al. (2015)
Cholangiocarcinoma	*In vitro*, *in vivo*	YAP	Induced EMT and cancer progression	Pei et al. (2015)
Cervical cancer	*In vitro*	YAP and TAZ	Induced proliferation, migration and invasion	Patterson et al. (2024)
Cervical cancer and colon cancer	*In vitro*	TAZ/YAP	Induced proliferation, migration and invasion	Arioka et al. (2025)
Glioblastoma	*In vitro*, *in vivo*	YAP/TAZ	Stimulated plasticity and stemness	Castellan et al. (2021)
	*In vivo*, clinical samples	YAP/TAZ	Enhanced tumorigenesis and malignant form of glioblastoma	Yang et al. (2023)
Hepatocellular carcinoma	*In vitro*, *in vivo*, clinical samples	YAP	Induced MDR through RAC1-ROS-mTOR pathway and repression of autophagy-mediated cell death	Zhou et al. (2019)
	*In vivo*	YAP/TAZ	Enhanced tumor growth	Moya et al. (2019)
	*In vitro*, clinical samples	YAP/TAZ	Tumor cell migration and invasion	Weiler et al. (2020)
Lung cancer	*In vitro*, clinical samples	YAP	Induced EMT, metastasis and cancer progression	Guo et al. (2017)
	Clinical samples	YAP	Enhanced tumor progression	Kim et al. (2011)
Osteosarcoma	*In vitro*, clinical samples	YAPl	Stimulated the proliferation and invasion through RUNX2 pathway	Zhang et al. (2013)
	Clinical samples	YAP/TAZ	Enhanced tumor progression	Bouvier et al. (2016)
Prostate cancer	*In vitro*, *in vivo*	YAP1	Enhanced tumor progression	Kuser-Abali et al. (2015)
	*In vitro*, clinical samples	YAP1	Modulatory effects on PSA expression through the AR	Wang et al. (2024)
	*In vitro*, *in vivo*	YAP	Augmented castration resistant growth as well as invasion and migration	Zhang et al. (2015)

The onset and progression of cancer are complex processes involving numerous elements. Abnormal modification in the hippo pathway are frequently observed in various cancer types in both human and animal models, including breast, colorectal, lung, brain, uveal and esophageal cancers, particularly related to dysregulation of YAP/TAZ/TEAD axis (Han et al., 2019a; Plouffe et al., 2015). For instance, the formation of glioma was linked to the of YAP/TAZ abnormal activation, which was caused by the downregulation of LATS1/2 (Zhang et al., 2016). A novel crosstalk mechanism between the Hippo/YAP and Wnt/β-catenin pathways has recently been identified, which plays a functional role in glioma proliferation (Wang et al., 2017). In hepatocellular carcinoma patients, upregulated activity of YAP/TAZ have been associated with aggressive molecular characteristics and poor outcome negative survival outcomes (Perra et al., 2014). Unlike YAP, there is a scarcity of studies examining the role of TAZ in various malignancies. However, Yang L. et al. (2021) indicated that nuclear transfer of TAZ is linked to an extremely aggressive triple-negative variant of breast carcinoma. Moreover, hyperactivation of TEAD and associated proteins were shown to exert a significant influence on carcinogenesis process. TEAD1 is known to be an important controlling element in the differentiation of prostatic epithelial cells and morphogenesis of epithelial cells. Higher levels of TEAD1 have been linked to worse prognosis in patients with prostate cancer (Shen et al., 2020; Song et al., 2024), while higher levels of TEAD2 can cause hepatocellular carcinoma (Joo et al., 2020). In addition to prevalent malignancies, other concurrent upregulation of YAP/TAZ has been documented to play crucial roles in the development of some uncommon cancers in a context-dependent manner. The TAZ-CAMTA1 fusion protein was detected in over 90% of patients with epithelioid hemangioendothelioma (EHE) because of highly prevalent gene mutation (translocation) (Suto, 2024). There are several ways in which upregulated activity of YAP/TAZ/TEAD complex promotes carcinogenesis. In many cancer cell lines, AP-1-linked gene expression process are important for cell cycle regulation, and the YAP/TAZ-TEAD complex is required for these programs to function properly. In addition, the complex can aberrantly control cell cycle related transcription factors like AP-1, leading to overproliferation, cellular migration, and metastasis (Zanconato et al., 2015). Also, hyperactive YAP/TAZ controls the transcription of metabolic genes that can increase the transport of glucose and amino acids that resulted in a metabolic change towards aerobic glycolysis and helps in tumor growth in situations with limited nutrients (Cox et al., 2018). Moreover, these abnormal metabolic pathways may subsequently facilitate YAP/TAZ activation, thereby promoting the onset and progression of many malignancies (Zhang et al., 2018).

Furthermore, YAP/TAZ is an important element of EMT, a mechanism that stimulates cells to detach from their surface and adapt an invasive and motile phenotype (Zhang et al., 2021). Epithelial-mesenchymal transition (EMT) facilitates the dissemination of cancer cells to distant sites, hence enhancing metastasis (Fedele et al., 2022). YAP/TAZ modulates EMT by modifying the expression of crucial proteins, including ZEB1/2, Twist, and Snail (Ichikawa et al., 2022). These interactions upregulated the level of mesenchymal markers (vimentin and N-cadherin) and downregulated the level of epithelial markers (E-cadherin) (Bullock and Brunton, 2025). Examination of cancer-specific YAP/TAZ signaling impacts on tumor aggressiveness and epithelial-mesenchymal transition. YAP/TAZ increases resistance to anticancer agents and augmented migratory potential in lung cancer (Franklin et al., 2023). The augmentation of stemness and plasticity in breast carcinomas by YAP/TAZ facilitates immune evasion and adaptation to adverse environments (Guo and Han, 2023). YAP/TAZ facilitates mechanotransduction in liver cancer, allowing cancer cells to adapt to heightened tissue stiffness, a significant characteristic of hepatocellular carcinoma (Xuan et al., 2025). Similar to ovarian and renal malignancies, dysregulation of YAP/TAZ serves as a prognostic indicator of poor outcomes, suggesting its role in metastatic advancement and resistance to pharmacological therapies (Piccolo et al., 2023).

However, several reports have suggested that they are not functionally redundant in cancer. Deletion of YAP or TAZ causes different phenotypes that cannot be rescued by the other, indicating specialized functions of each regulator (Plouffe et al., 2018). Further genome-wide studies have revealed that YAP and TAZ regulate overlapping but distinct sets of gene, pointing to differences in how they select enhancers and recruit other regulatory cofactor (Reggiani et al., 2021). In cancer, YAP primarily drives tumor growth and proliferation. In contrast, TAZ is more strongly linked to increased cell invasion, epithelial-mesenchymal transition, and cancer stem cell traits, particularly in advanced tumors (Shreberk-Shaked and Oren, 2019). These findings collectively show that YAP and TAZ are distinct carcinogenic regulators whose roles depend on biological context.

Recent research has elucidated new aspects of the Hippo pathway, particularly the role of YAP/TAZ/TEAD in carcinogenesis, thereby creating new opportunities for therapeutic interventions. The information gathered from these investigations offers fresh perspectives on the intricacy of cancer and may result in development of more treatment methods that specifically target the Hippo pathway (Yu et al., 2025).

## 4 Anticancer natural compounds as modulators of hippo signaling

**FIGURE 2:**
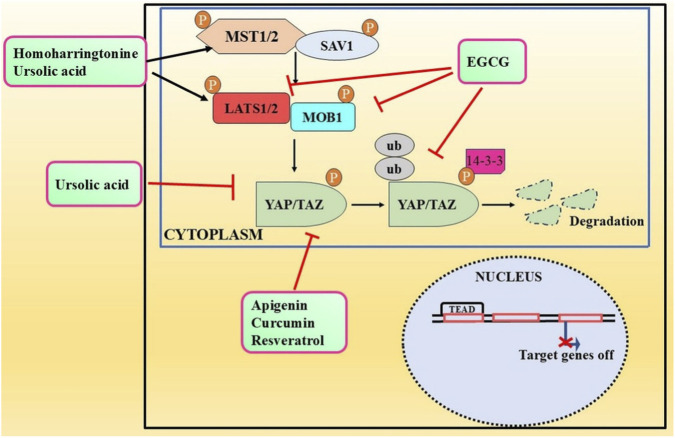
Modulatory effects of plant derived anticancer compounds (apigenin, curcumin, EGCG, resveratrol, homoharringtonine, and ursolic acid) on MST1/2, LATS1/2, MOB1, YAP and TAZ proteins of signaling pathway.

The Hippo signaling is crucial in regulating organ size and tumorigenesis by suppressing cell proliferation, promoting apoptosis, and modulating the development of stem cells (Barry et al., 2021). The accumulation of unphosphorylated YAP/TAZ in the nucleus results in tumor growth and development. The cytoplasm serves as the primary site for phosphorylated YAP/TAZ, inhibiting cancer proliferation. There is a correlation between various malignant tumors and enhanced activity and expression of YAP/TAZ (Mohajan et al., 2021). The advancement of targeted Hippo pathway interventions for cancer chemoprevention and treatment using natural chemicals remains in its nascent phases, although it holds significant promise for the future (Mohammadi et al., 2020; Charoenwongpaiboona et al., 2021). Apart from other studies, we therefore examined recent advancements in the application of extremely effective anticancer phytochemicals (apigenin, curcumin, EGCG, resveratrol, homoharringtonine, and ursolic acid) to target Hippo components in cancer treatment (Table 2) (Figure 2).

**TABLE 2:** Effects of various plant derived anticancer compounds on cellular and molecular targets of Hippo signaling pathway in different types of carcinomas.

Anticancer compound	Class	Cancer type	Cancer model	Target	References
Apigenin [C_15_H_10_O_5_ 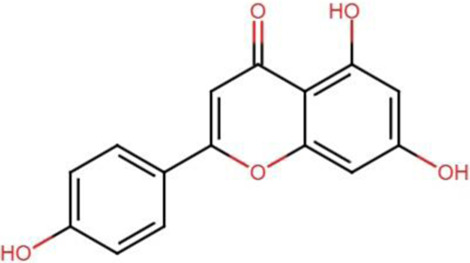	Polyphenols	Hepatocellular carcinoma	SMMC-7721 and SK-Hep1 cells	Downregulated expression of YAP	Zeng et al. (2022)
	Polyphenols	Breast cancer	MDA-MB-231 cells	Downregulated activity of YAP/TAZ and transcriptional activation of CTGF and CYR61 genes	Li Y. W. et al. (2018)
Curcumin [C_21_H_20_O_6_] 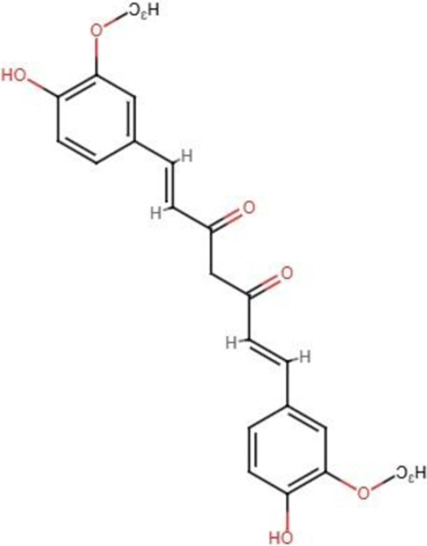	Polyphenols	Pancreatic cancer	Patu8988 and Panc-1 cells	Downregulated expression of YAP/TAZ	Zhou et al. (2016)
	Polyphenols	Colon cancer	HCT116 and SW620 cells	Downregulated expression of YAP	Zhu et al. (2018)
	Polyphenols	Bladder cancer	5637 and WH cells	Downregulated YAP/TAZ level	Gao et al. (2014)
	Polyphenols	Lung cancer	A549, NCI-H1299 cells	Induced TAZ protein degradation	Zheng et al. (2021)
Epigallocatechin-3-gallate [C_22_H_18_O_11_] 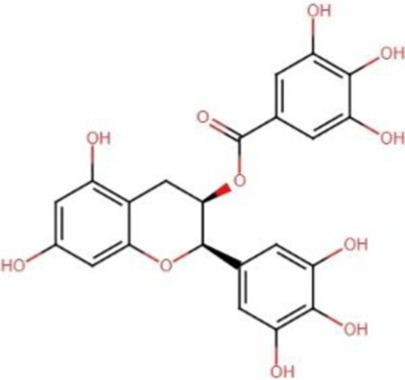	Polyphenols	Tongue squamous cell carcinoma	CAL27 and SCC15	Downregulated expression of TAZ, LATS1 and MOB1	Li A. et al. (2018)
Resveratrol [C_14_H_12_O_3_] 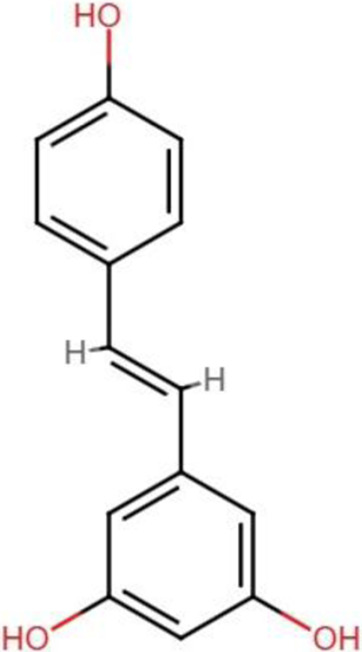	Polyphenols	Colon cancer	HCT116 cells	Downregulated expression of I YAP and its downstream targets CTGF and CYR61	Qin et al. (2022)
	Polyphenols	Gastric cancer	SGC-7901 cells	Downregulated expression of YAP	Deng et al. (2022)
	Polyphenols	Thyroid cancer	Nthy-ori 3-1 and FTC133 cells; FTC238 cells xenograft	Downregulated expression of YAP/TAZ	Xu et al. (2020)
Homoharringtonine [C_29_H_39_O_9_] 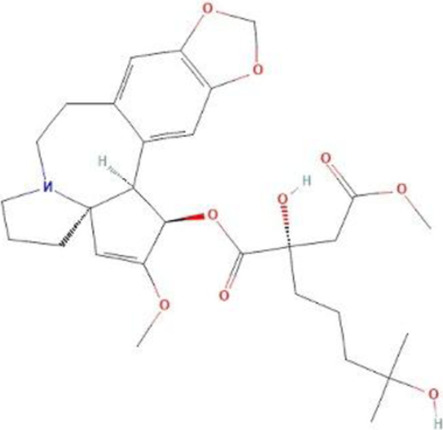	Alkaloid	Hepatocellular carcinoma	HepG2, Huh7, SMMC-7721 cells	Upregulated phosphorylation of MST1, MST2, MOB1, LAST1, and YAP	Wang et al. (2021)
Ursolic acid [C_30_H_48_O_3_] 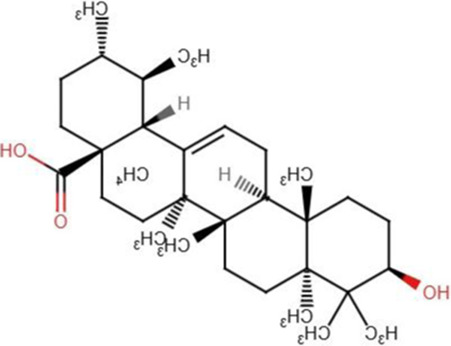	Terpenoid	Gastric cancer	SNU484 and SNU638 cells	Upregulated protein expression of RASSF1, MST1, MST2, LATS1, and while YAP1, CTGF FOXM1, KRAS, and BATF expression decreased	Kim et al. (2019)

### 4.1 Apigenin

Apigenin is a naturally occurring flavonoid that is classified under the flavone category of glycosides. It is commonly found in various vegetables, fruits, and herbs such as oranges, grapefruit, onions, parsley, chamomile and basil (Salehi et al., 2019). A number of studies have shown that apigenin may have antioxidant and anti-inflammatory effects which makes it an attractive candidate for lowering the risk of pathogenesis (Lee et al., 2023; Charrière et al., 2024; Lu et al., 2023). Additionally, apigenin has been demonstrated to boost the internal defense against oxidative stress by increasing the function of key enzymes (Gao et al., 2012). Based on the results of multiple studies, this phytochemical has the potential to suppress angiogenesis, invasion and migration, induce apoptosis and cell cycle arrest which ultimately suppress a variety of cancers (Zhang et al., 2024; Bhagavatula et al., 2024; Pandey et al., 2023; Golonko et al., 2024). Furthermore, apigenin exhibit antitumor effects through upregulation of caspases, Bax, p53, and TNF-α and the downregulation of Bcl-2, cyclins, CDKs and MMP-2/-9 (Liang et al., 2022). It is also involved in the modulation of key intermediates of signaling pathways, such as the reduction of PI3K/AKT, mTOR, NF-κB, JNK, STATs, β-catenin, and Notch1 levels (Maashi et al., 2022; Chen et al., 2022).

Apigenin therapy suppressed the viability, migration, and invasion of HCC SMMC-7721 and SK-Hep1 cells *in vitro*. It diminished YAP expression, thereby decreasing migration and invasion by altering EMT indicators, and enhanced autophagy in liver cancer cells by regulating autophagy-related gene expression (Zeng et al., 2022). Mechanistic investigation revealed that apigenin markedly inhibited the proliferation and migration of TNBC cells. In breast cancer cells, apigenin treatment downregulated the activity of YAP/TAZ as well as the expression of CYR61 and CTGF target genes. Moreover, apigenin inhibited the interaction of YAP/TAZ/TEAD proteins and reduced TAZ expression, thereby sensitizing breast cancer cells to apigenin therapy (Li Y. W. et al., 2018).

### 4.2 Curcumin

Curcumin has demonstrated anti-inflammatory, antioxidant, antibacterial, antitumor, and antimutagenic activities, making it as suitable supplement for various ailments. Numerous reports from the past few decades have suggested that curcumin has strong potential against multiple types of cancer (Amaroli et al., 2024). It regulates a variety of growth regulators, transcription factors, inflammatory factors (cytokines), and cell signaling molecules, and hence inhibits the growth and spread of different forms of cancers (Kunnumakkara et al., 2008). Curcumin has attracted considerable interest because of its possible anticancer effects, both independently and in conjunction with chemotherapeutic drugs. Recent research has emphasized its capacity to impede multiple phases of carcinogenesis, including angiogenesis, tumor promotion, tumor growth (Parker et al., 2025).

A molecular-based investigation showed that curcumin therapy markedly decreased cell proliferation, diminished clonogenic potential, obstructed migration and invasion, and triggered cell cycle arrest and apoptosis in pancreatic cancer cells. In addition to this, curcumin significantly reduced the expression of YAP and TAZ, which in turn lowered the expression of Notch-1 (Zhou et al., 2016). Zhu et al. investigated whether YAP serves as a target of curcumin in colon cancer cells. The results demonstrated that curcumin suppressed cell growth and triggered autophagy in colon cancer cells. It was observed that the expression of YAP was decreased following treatment with curcumin. Furthermore, autophagy was suppressed upon overexpression of YAP, whereas a reduction in YAP expression triggered autophagy. This study suggests that curcumin could be a good treatment for colon cancer since it might be able to stop YAP and reverse autophagy (Zhu et al., 2018).

A therapeutic investigation of curcumin demonstrated that it facilitates the proteasome-dependent degradation of KLF5 by targeting the YAP/TAZ pathway in bladder cancer cells. This study revealed that lentivirus-mediated knockdown of KLF5 suppressed cancer cell proliferation, whereas overexpression of Flag-tagged KLF5 partially counteracted the curcumin effects on cell proliferation and cyclin D1 level. Moreover, curcumin may inhibit the production of Hippo pathway effectors, YAP and TAZ, which are known to safeguard KLF5 from destruction (Gao et al., 2014). Zheng et al. conducted additional research to understand the mechanistic role of curcumin against lung cancer cells by focusing on elements of the hippo pathway that are significantly important. Mechanistically, curcumin has been shown to facilitate the nuclear-cytoplasmic translocation of TAZ, but not YAP, which are the crucial elements of the Hippo pathway. Furthermore, curcumin impaired TAZ protein stability and facilitated TAZ protein breakdown in lung cancer cells, relying on the proteasome degradation system rather than the autophagic-lysosomal degradation route. When TAZ was overexpressed, it restored the ability of curcumin to suppress the stemness of lung cancer cells (Zheng et al., 2021).

### 4.3 Epigallocatechin gallate

Among the several flavonoids present in green tea is epigallocatechin gallate, more commonly known as EGCG. Extensive research has been conducted on EGCG to investigate its possible health benefits, particularly in the context of cancer. Preclinical experimental evidences suggests that EGCG may have growth inhibitory, anti-angiogenic, and apoptotic inducing effects in various cancer models (Almatroodi et al., 2020). EGCG can disrupt many signaling pathways linked to cellular proliferation and division across distinct cancer types. An increasing amount of evidence indicates that EGCG may address many cancer hallmarks, which are the essential biological processes and characteristics that facilitate cancer formation and progression (Farooqi et al., 2020; Talib et al., 2024). A plethora of *in vitro* investigations have demonstrated the efficacy of EGCG in attenuating proliferation, inducing apoptosis, and obstructing the migration and invasion of tongue squamous cell carcinoma cell lines *via* several molecular signaling pathways (Ch et al., 2012; Kanlaya et al., 2016; Shin et al., 2016). Li et al. investigated the potential links between EGCG stimulation and TSCC cell Hippo-TAZ signaling pathway activation in this regard. EGCG suppressed the proliferation of TSCC CAL27 and SCC15 cells by reducing the expression of LATS1, MOB1, TAZ and JNK at protein levels. Upregulation of TAZ reduced the effect of EGCG molecule in TSCC CAL27 cells. Furthermore, the combinatorial effect of EGCG and simvastatin significantly decreased cell growth, invasion and migration, while promoting apoptosis in CAL27 cells as compared to individual EGCG treatment (Li A. et al., 2018).

### 4.4 Resveratrol

Resveratrol, a natural bioactive compound found in grapes, red wine, and many other plant sources, has garnered considerable attention for its antineoplastic capabilities (Meng et al., 2020). The diverse range of cellular and molecular pathways involved in these features render resveratrol a promising anticancer agent against various malignancies such as breast, cervical, prostate, lung, and gastrointestinal cancers. In order to exert its anti-carcinogenic effects, resveratrol interacts with a wide variety of cellular signaling pathways that are associated with a wide variety of tumorigenesis processes, including cell proliferation, apoptosis, angiogenesis, cell cycle regulation, and metastasis (Almatroodi et al., 2022). Resveratrol also shown to inhibits oncogenic genes, stimulated tumor suppressing genes and modify tumor microenvironment (Peng and Jiang, 2018; Han et al., 2019b). Resveratrol also suppresses key oncogenic signaling pathways such as PI3K/AKT/mTOR, NF-κB, and STAT3 (Varoni et al., 2016).

A study by Qin et al. (2022) found that resveratrol treatment significantly decreases cell proliferation and causes cell cycle arrest and apoptotic induction in HCT116 cells. Further mechanistic exploration studies revealed that resveratrol downregulated the YAP protein level and downstream targets CTGF and CYR61 gene expression. Molecular docking analysis suggested a strong interaction of resveratrol with YAP-TEAD. Deng et al. (2022) also explored the antitumor efficacy of resveratrol by targeting Hippo-YAP signaling pathways in gastric cancer SGC-7901 cells. The results shown that resveratrol inhibited cell proliferation, migration and EMT of SGC-7901 cells. A downregulated expression of YAP was also reported in resveratrol treated- SGC-7901 cells, which resulted in deactivation of the Hippo-YAP pathway (Deng et al., 2022). Moreover, Xu et al. demonstrated that resveratrol may impede follicular thyroid cancer *via* a mechanism associated with direct interaction with ST6GAL2 and the Hippo pathway. The findings suggested that ST6GAL2 expression was downregulated in follicular thyroid carcinoma following resveratrol treatment as compared to control cells, implying resveratrol plays a role in this mechanism (Xu et al., 2020).

### 4.5 Homoharringtonine

Homoharringtonine (HHT) is a plant-derived alkaloid which is known for its antitumor efficacy. Recent research has also shown that HHT may have neuroprotective capabilities, in addition to its immunomodulatory, antiviral and antifibrotic effects. HHT has exhibited remarkable potential in clinical studies for the treatment of hematological cancers specifically acute and chronic myeloid leukemia (Khatua et al., 2024; Swerdlow et al., 2016). Additionally, antitumor efficacy of HHT against CML has been established by multiple studies performed in the United States (Quintás-Cardama et al., 2009). A variety of diseases and conditions have shown HHT to be an effective anticancer agent; these include acute promyelocytic leukemia, polycythemia vera, acute promyelocytic leukemia, and central nervous system leukemia (Kantarjian et al., 2013). Its proven antiproliferative effects on various tumor cells including breast, skin, and gastrointestinal cancers (Tang et al., 2021; Guo et al., 2021).

In hepatocellular carcinoma cells, HHT markedly suppressed tumor cell growth, migration and invasion, as well as colony forming ability by activating the Hippo pathway. HHT exposure resulted in elevated rate of phosphorylation of major key proteins of the Hippo pathway including YAP, MST1/2, and MOB1. Moreover, HHT treatment leads to increased expression of SAV1. Overall, this study suggested that the Hippo pathway mediates the growth inhibitory role of HHT in hepatocellular carcinoma (Wang et al., 2021).

### 4.6 Ursolic acid

Ursolic acid is a pentacyclic triterpene that is extracted from numerous vegetables, fruits, and various traditional medicinal herbs (Arulnangai et al., 2025). Ursolic acid has been widely utilized in cancer therapy in Traditional Chinese Medicine (TCM) for numerous years. Over the past two decades, ursolic acid has been evaluated for its efficacy in preventing cancer progression and as a therapeutic intervention for several malignant tumors. Most studies have focused on its effects on energy metabolism, cellular proliferation, and antioxidant activities (Nguyen et al., 2021; Prasad et al., 2016; Kim S. H. et al., 2015; Chen et al., 2020). These preclinical investigations have demonstrated that ursolic acid decreases various aspects of cancer cell proliferation, energy metabolism, and inflammation generated by tumor cells (Wang et al., 2013; Kim et al., 2014; Zhao et al., 2021; Zafar et al., 2022).

Kim et al. revealed the mode of action of ursolic acid in gastric cancer cells by modulating the Hippo pathway. Their findings shows that the colony counts and dimensions of cancer cells were significantly reduced following the administration of ursolic acid. Ursolic acid also repressed the invasion and migratory rates of these cells. The findings of gene ontology analysis indicated that several key intermediates of Hippo pathway were effectively altered by ursolic acid including MST1, MST2, YAP1, and LATS1. Furthermore, the protein expression analysis were in accordance with the findings of gene ontology analysis. In xenograft tumor model, ursolic acid treatment resulted in elevated levels of Hippo pathway-associated proteins, thus demonstrating its potential to inhibit gastric tumors *in vivo* (Kim et al., 2019).

## 4 Limitations

The use of plant derived compounds has demonstrated significant promise in cancer therapy due to their diverse mechanisms, which include antioxidant, antiproliferative and immunomodulatory effects (Ai et al., 2024). Many phytochemicals possess notable anticancer properties, however they often lack drug like characteristics, obstructing their clinical application. A major limitation is their poor aqueous solubility with over 40% of plant derived compounds exhibiting insufficient aqueous solubility, which restricts their absorption in gastrointestinal tract and reduces systemic circulation (Nicolaescu et al., 2025). Additionally, once absorbed, many phytochemicals undergo swift first pass metabolism in the liver and intestines, leading to quick degradation and elimination from the body (Devaraji and Thanikachalam, 2025). The unstable nature of these natural compounds such as curcumin and quercetin, further decreases their bioavailability before they reach their target sites (Aljabali et al., 2025). Limited membrane permeability and non-specific distribution hinder their accumulation at tumor sites, hence increasing the risk of adverse effects in nearby areas. The medicinal potential of these phytochemicals is constrained by challenges related to solubility, stability and bioavailability. To address these challenges and fully harness the therapeutic benefits of phytochemicals, it is essential to develop better delivery carriers that can protect them, enhance their absorption and enable targeted actions (Peñaherrera-Pazmiño et al., 2025).

## 5 Conclusion and future trends

Recently, numerous insights into the Hippo pathway have explored its mechanism of activation, crucial regulators, and upstream and downstream targets, thereby enhancing our understanding of the involvement of this pathway in several types of carcinomas. Thus, targeting this pathway and its regulators could be a promising strategy for anticancer therapy. This review highlights the recent research reports on key anticancer phytochemicals, including apigenin, curcumin, EGCG, resveratrol, homoharringtonine, and ursolic acid that modulate the Hippo-YAP/TAZ signaling pathway. In most cancer types, altered expression of YAP and TAZ is associated with drug resistance, tumor growth, metastasis, and EMT. Considering the potential role of Hippo-YAP/TAZ signaling, efforts are being made to discover various agents that target this pathway. In this context, the application of phytochemicals has demonstrated significant potential in inhibiting cancer cell growth by targeting YAP/TAZ and other regulators of the Hippo pathway. However, research focusing on this pathway is in its early stages, and the majority of the natural compounds examined in this review have been investigated in preclinical studies. While preclinical experimental studies have provided insights into the influence of phytochemicals on Hippo-YAP/TAZ signaling pathway, there is still a need for a more profound understanding of how these compounds modulate these factors, consequently enhancing their therapeutic efficacy. The main goal of future research should be to transform preclinical results into clinical outcomes. Further exploration of the mechanisms behind natural compounds-based targeting of Hippo pathway could yield valuable insights and potentially open the new avenue to innovative combinatorial therapies, improved drug delivery methods that explore the unique properties of these compounds.
